# The Epigenetic Repertoire of *Daphnia magna* Includes Modified Histones

**DOI:** 10.1155/2012/174860

**Published:** 2012-04-04

**Authors:** Nicole F. Robichaud, Jeanette Sassine, Margaret J. Beaton, Vett K. Lloyd

**Affiliations:** Department of Biology, Mount Allison University, Sackville, NB, Canada E4L 1G7

## Abstract

Daphnids are fresh water microcrustaceans, many of which follow a cyclically parthenogenetic life cycle. *Daphnia* species have been well studied in the context of ecology, toxicology, and evolution, but their epigenetics remain largely unexamined even though sex determination, the production of sexual females and males, and distinct adult morphological phenotypes, are determined epigenetically. Here, we report on the characterization of histone modifications in *Daphnia*. We show that a number of histone H3 and H4 modifications are present in *Daphnia* embryos and histone H3 dimethylated at lysine 4 (H3K4me2) is present nonuniformly in the nucleus in a cell cycle-dependent manner. In addition, this histone modification, while present in blastula and gastrula cells as well as the somatic cells of adults, is absent or reduced in oocytes and nurse cells. Thus, the epigenetic repertoire of *Daphnia* includes modified histones and as these epigenetic forces act on a genetically homogeneous clonal population *Daphnia* offers an exceptional tool to investigate the mechanism and role of epigenetics in the life cycle and development of an ecologically important species.

## 1. Introduction

Daphnids are freshwater crustaceans that hold the distinction of being among the relatively few genera that reproduce parthenogenetically. Under most circumstances conventional oogenesis is modified. The first meiotic division is abortive so only the mitosis-like equational division occurs producing clonal diploid eggs [1, 2]. While homologs do pair in the abortive first meiotic division [2] and many of the same meiotic genes are expressed in parthenogenetic and sexual reproduction [3], there is no cytological [2] or genetic [3, 4] evidence for recombination. As a result, other than rare mitotic recombination, conversion, or mutational events [5], the progeny produced are genetically identical [1, 2, 4]. However, while the offspring are genetically identical to each other and their mother, they are not necessarily epigenetically identical. Under stressful conditions some of these clonal diploid eggs develop as males rather than females [1, 6–8]. Additionally, in many species stressful conditions similarly trigger the restoration of conventional meiosis allowing production of haploid eggs and sperm [1–3, 6, 8]. Importantly, parthenogenetically reproducing females and sexually reproducing females are genetically identical, and both are identical to their mothers [1, 4, 5]. Moreover, parthenogenetically produced males are genetically identical to parthenogenetically produced females [1, 4, 5]. Thus, environmental signals induce epigenetic changes that control essential aspects of the life cycle—sex determination and sexual reproduction.

Epigenetic variation in daphnids has also been studied in the context of environmentally induced morphological changes, which are termed polyphenisms. In the presence of predators, *Daphnia* can produce a variety of defensive structures such as helmets, neckteeth, crests, or elongated tail spines and spikes, depending on the species [9]. As these changes occur in parthenogenetic populations in which all animals are genetically identical clones, these changes are necessarily epigenetic [9–11].

Although *Daphnia* provide an excellent system for the study of epigenetics, surprisingly, this system has not been widely exploited. This is despite the rich literature relating to their evolution, reproduction, and ecology. There are also many genomic tools available for studying these organisms, including the genome sequence of *D. pulex* [12, 13], which has allowed the development of bioinformatic and other genomic technologies such as microarrays [14, 15], cytogenetics [16], cell culture [17], transgenics [18, 19], and RNAi gene knockdown technology [19]. *Daphnia* are ubiquitous and key members of aquatic communities, a role that has led to their extensive use in ecotoxicology, and more recently ecotoxicogenomics [15]. Because of the ecological importance of daphnids as well as their unusual development, understanding their epigenetic repertoire and its deployment in normal development and under environmental stresses is significant, yet the epigenetic resources of daphnids, which is how the environment regulates the genome, remain poorly explored. 

Investigations into *Daphnia* epigenetics, to date, have focused primarily on DNA methylation. Partial sequencing of the *D. magna* genome revealed that this species has homologs of the three major vertebrate DNA methyl transferases, Dnmt1, Dnmt2, and Dnmt3A [20] and that CpG methylation does occur [21]. While the level of methylation is relatively low, it is sensitive to developmental stage, increasing modestly in adults from 0.13% of all CpG dinucleotides in 7-day-old individuals to 0.26% in 32-day-old individuals [21]. Investigation of other core epigenetic processes such as histone modification or noncoding RNA, or the role of these epigenetic mechanisms in either normal development or the well-studied predator-induced epigenetic polyphenisms, has yet to be pursued. Here, we report that *D. magna* shows both histone H3 and H4 modifications in embryonic cells. Furthermore, one of these modifications, histone H3 dimethylated at lysine 4 (H3K4me2), occurs nonuniformly in a cell-cycle-specific manner in gastrula cells and is absent from oocytes.

## 2. Materials and Methods

### 2.1. *Daphnia magna* Culture

*Daphnia magna* were acquired from WARD's Natural Science. They were kept at room temperature (25 ± 5°C) in 150 mL cups filled with synthetic pond water and fed with 2-3 mL of *Scenedesmus* culture (WARD's Natural Science) three to four times weekly. The algae were grown at 20°C in twenty-four hours of light in Bold's Basal Medium.

### 2.2. Histone Protein Analysis by Immunohybridization

80 young embryos were rapidly dissected from the mother's brood pouch in 0.6% NaCl and 0.03% triton X-100 and stored in 1.5 mL microtubes on ice for no more than 30 min. The liquid was removed and replaced with 200 *μ*L of 0.05 M DTT and 1X NuPAGE LDS Sample buffer (Invitrogen). The embryos and loading buffer were heated at 96°C for 5 min. and cooled to room temperature and the solution collected by centrifugation for 10 sec. 15 uL of the homogenate was electrophoresed on a 4–12% SDS-PAGE gel (Invitrogen) at 200 V for 40 min. 2.5 *μ*L Precision Plus Protein Standards (Bio-Rad) and Magic Mark (Invitrogen) were used as molecular weight standards. Gels to be immunoblotted were transferred to a PVDF membrane (Bio-Rad) in an XCell II chamber (Invitrogen) at 30 V for 80 min. The membrane was incubated in 2% Enhanced-Chemiluminescence (ECL) blocking agent (Amersham) in 0.1% TBST (5X; 12.1 g TRIS, 40 g NaCl, pH 7.6 with HCl) for 15 min at room temperature, followed by 15 ± 5 h at 4°C. The blocking agent was removed, and 10 mL of diluted primary antibody in 2% ECL with 0.1% TBST was added to the membrane and incubated for 60 ± 2 min at room temperature. The primary antibodies (mouse monoclonal antibody to histone H3 trimethyl K27 (H3K27me3; Abcam 6002), rabbit polyclonal antibody to histone H4 dimethyl K20 (H4K20me2; Abcam 9052), rabbit monoclonal antibody to histone H3 acetyl K14 (H3K14ac; Abcam 52946), rabbit monoclonal antibody to histone H3 dimethyl K4 (H3K4me2; Abcam 32356), or rabbit polyclonal antibody to histone H3 monomethyl K9 (H3K9me; Abcam 9045)) were diluted 1 : 500. The membrane was washed with 0.1% TBST twice for 3 sec, once for 15 min, and thrice for 5 min. 10 mL of secondary antibody (1/3,000 dilution of goat polyclonal to rabbit IgG, HRP conjugated (Abcam)) was added and incubated for 60 ± 10 min. The membrane was washed twice for 3 sec, once for 15 min, and thrice for 5 min with 0.1% TBST, developed with Lumigen developing reagent (Amersham) for 5 min with minimal light exposure and imaged with a Fluor-S-Imager (Bio-Rad).

### 2.3. Collection and Staging of *Daphnia* Embryos for *In Situ* Immunodetection

Immunocytology was performed, with some modifications, using the procedure employed in [22] and kindly provided by Y. Shiga. For convenience, the procedure is described below. Embryos or ovaries were dissected from adults using a fine tip probe (Moria Instruments) and placed in 1.5 mL of 0.6% NaCl and 0.03% Triton X-100 in 1.5 mL microtubes. Stage 1 and 2 embryos were selected for dissection based on their size, colour, and other morphological characteristics as outlined in [23]. The ovary was collected by removing the carapace and separating the ovary from the gut with a fine tipped probe. 

After collection of embryos, the NaCl Triton X-100 solution was removed and replaced with 1.5 mL of a 3 : 1 ratio of 1.33X phosphate-buffered saline (PBS) and 37% formaldehyde and 50 mM EGTA. The samples were allowed to fix for 20 minutes at room temperature. The fixative was removed by pipet, and samples were washed (for all washes 1.5 mL of the solution was added, left for 5 minutes, removed by pipet, and replaced with another 1.5 mL of solution) sequentially with 25%, 50%, 75%, and 100% methanol. The samples were then frozen in 1.5 mL of 100% methanol at −20°C. 

The samples were brought to room temperature and then washed five times with 100% methanol. Samples were then washed five times with 1X phosphate-buffered saline and 0.1% polysorbate 20 (PT). Samples were then washed three times with 0.1 M Tris, 0.15 M NaCl, and 0.5% bovine serum (TNB). For mechanical lysis of the vitelline and other embryonic membranes, embryos were subjected to three freeze/thaw cycles in which the embryos, in 500 *μ*L TNB, were frozen at −80°C for 30 min and then rapidly brought to room temperature. Ovaries were not subjected to freeze/thaw cycles. Samples were then washed twice more in 1.5 mL TNB and left in 1.5 mL TNB for 1 hour. A 1 : 10 dilution of the primary antibodies was added to the samples in 49 *μ*L of TNB, making a final dilution of 1 : 500, and the samples were incubated at 4°C overnight. The solution containing the primary antibody was then removed and the samples washed five times in TNB. 1 uL of the secondary antibody, goat anti-rabbit IgG FITC conjugate (Zymed), was added to 49 *μ*L of TNB and embryos and incubated for 2 hours at 4°C in the dark. The samples were protected from light for the remainder of the experiment. The samples were washed five times with TNB and then five times with 0.1 M Tris, 0.15 M NaCl, and 0.05% polysorbate 20 (TNT). 15 *μ*L of 10 *μ*g/mL 4′-6-diamidino-2 phenylindole (DAPI) in TNT was added to the samples in 1.5 mL TNT and left for five minutes at room temperature. Excess DAPI solution was removed and the samples washed twice for 5 min with TNT.

To visualize the samples, excess TNT was removed and embryos or tissues were placed in a drop of Vectashield mounting medium (Vector Labs) before adding a cover slip. The embryos or tissues were viewed using an Axioscope 2 Plus (Zeiss) fluorescent microscope. Images were captured with Axiovision AC software (Zeiss). 

## 3. Results

Histone modifications are one of the most important and conserved aspects of epigenetic gene regulation [24–27] and as such are a good target for an initial investigation into *Daphnia* epigenetics. Further, histone proteins are among the most highly conserved proteins in eukaryotes [28] so it seemed likely that commercially available antibodies raised to modified histones in other species would also work in *Daphnia*. 

### 3.1. Confirmation of Antibody Specificity

**Figure 1:**
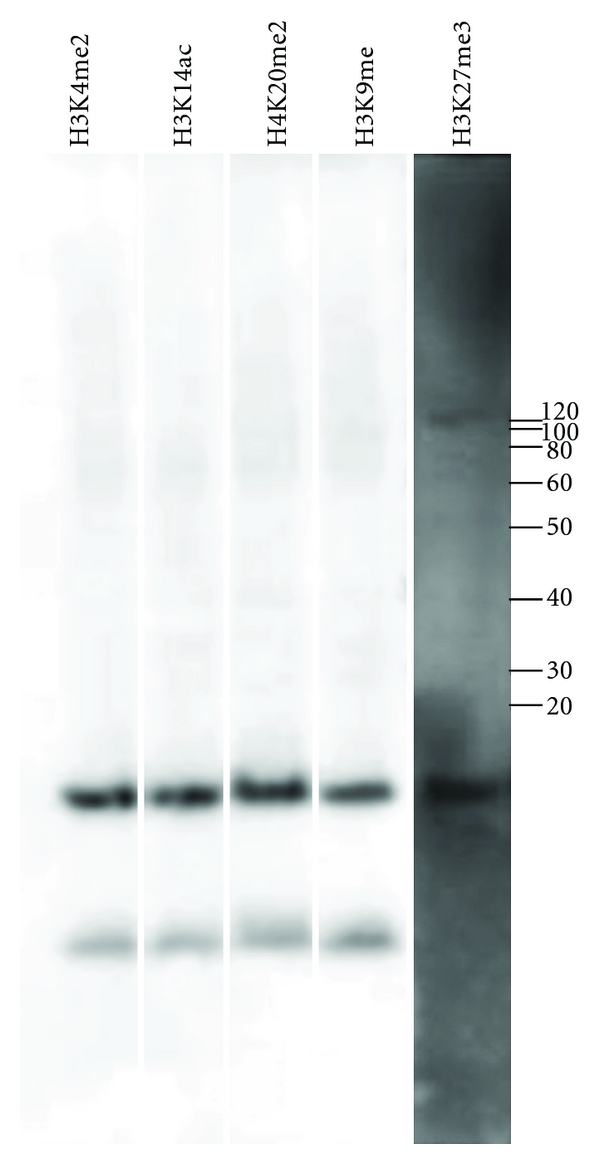
Immunohybridization of *Daphnia magna* embryos with antibodies specific to modified histone H3 and H4. Immunohybridization of protein extracted from *Daphnia* embryos with antibodies to histone H3 trimethyl K27 (H3K27me3), histone H4 dimethyl K20 (H4K20me2), histone H3 acetyl K14 (H3K14ac), histone H3 dimethyl K4 (H3K4me2), and histone H3 monomethyl K9 (H3K9me). These antibodies all detect a strong band at 17 kDa, the expected size for histone H3 and histone H4.

To confirm this supposition, we performed immunohybridization of *Daphnia magna *embryos with antibodies against human histone H3 modified by trimethylation of lysine 27 (H3K27me3), dimethylation of lysine 4 (H3K4me2), monomethylation of lysine 9 (H3K9me), acetylation of lysine 14 (H3K14ac) or histone H4 modified by dimethylation of lysine 20 (H4K20me2). As expected, these antibodies all detected a predominant band at 17 kD, the expected size of histones H3 and H4 (Figure 1). Weakly hybridizing bands at approximately 15 kDa and 100 kDa were also detected, particularly when the immunoblots were overexposed. Information from the supplier indicates that the H3K27me3 antibody detects a 15 kDa band from human cells, suggesting that this band represents histone protein fragments. The 100 kDa band likely represents proteins associated with cell and body fragments not completely removed during protein preparation.

### 3.2. Immunocytological Analysis of Blastula and Gastrula Embryos

**Figure 2:**
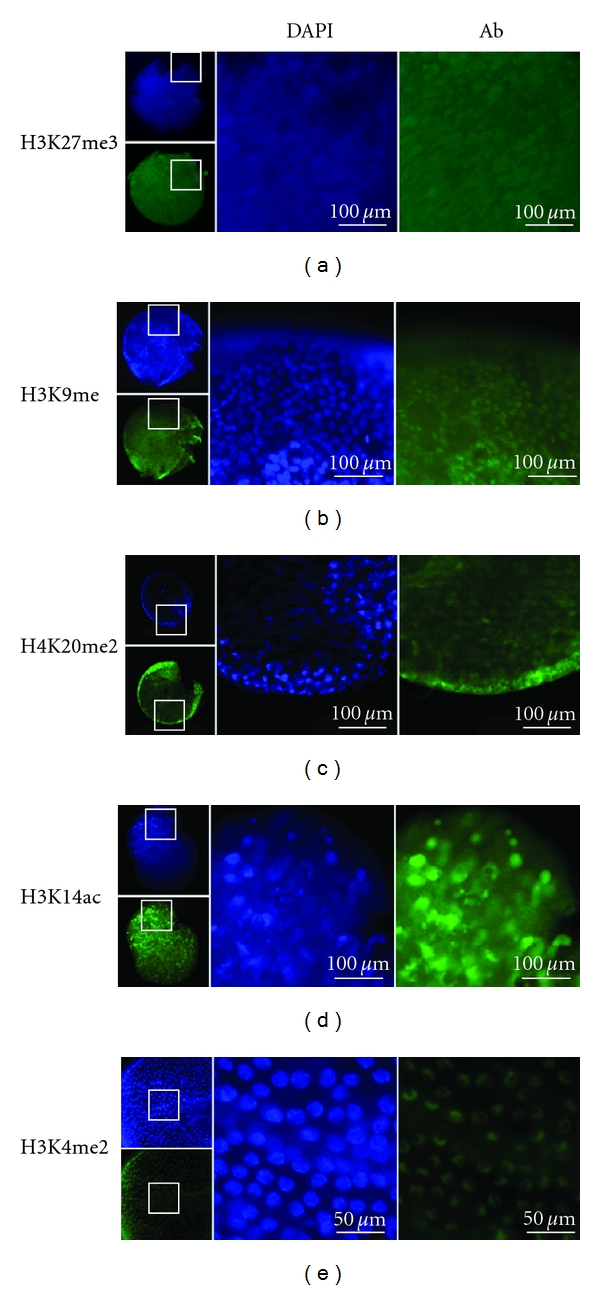
Whole mount immunocytochemistry of *Daphnia magna* embryos stained with antibodies specific to modified histone H3 and H4. The small images show the embryo from which the magnified images to the right are shown. Blue staining is with DAPI, which detects all DNA. Green staining (Ab) is for the specified histone modification. (a) H3K27me3. (b) H3K9me. (c) H4K20me2. (d) H3K14ac. (e) H3K4me2. The embryos shown in (a–d) are blastula stages, (e) is a magnified view of a gastrula embryo. The low magnification views show the torn extraembryonic membranes required to allow antibody penetration to the cells.

As the antibodies appear to detect the appropriate modified histones in *Daphnia*, we next used them to examine embryos for the presence and nuclear distribution of these modifications (Figure 2). To ensure that the antibodies were able to access the embryonic cells, the extraembryonic membranes were ruptured by freeze-thaw cycles, as described in Section 2, so that the normally spherical embryos show torn membranes and, occasionally, released embryonic cells.

Histone 3 trimethylation of lysine 27 (H3K27me3) and monomethylation of lysine 9 (H3K9me) are considered markers of heterochromatin [27, 29]. Dimethylation of lysine 20 of histone 4 (H4K20me2) has been shown to prevent acetylation at lysine 16 that would, in the absence of H4K20me2, promote the formation of euchromatin. Thus, the H4K20me2 modification indirectly promotes heterochromatin formation [27]. Antibodies specific to H3K27me3 and H3K9me (Figures 2(a) and 2(b)) show uniform nuclear staining, coinciding exactly with the DNA detected by DAPI staining. Similarly, H4K20me2 staining (Figure 2(c)) is uniform throughout the nucleus. This pattern was observed in multiple blastulae and gastrulae cells thus it appears invariant in these embryonic stages. 

Acetylation of histone H3 at lysine 14 (H3K14ac) and dimethylation of lysine 4 (H3K4me2) are considered to be markers of open or euchromatic chromatin [30]. Antibodies specific to H3K14ac also showed uniform staining of the nucleus that coincides with DNA staining by DAPI (Figure 2(d)). 

In contrast, staining with anti-H3K4me2 was consistently nonuniform with concentration at the nuclear periphery that did not completely coincide with DNA staining by DAPI (Figure 2(e)). The preferential staining of the nuclear periphery by H3K4me2 was not an artifact of antibody accessibility or interference from the various embryonic membranes as it was observed only with this antibody (Figure 2) and was also apparent in isolated cells released from the embryonic membranes by sonication (data not shown). The non-uniform distribution of H3K4me2 was reproducibly observed in cells from late blastulae to gastrulae embryos.

**Figure 3:**
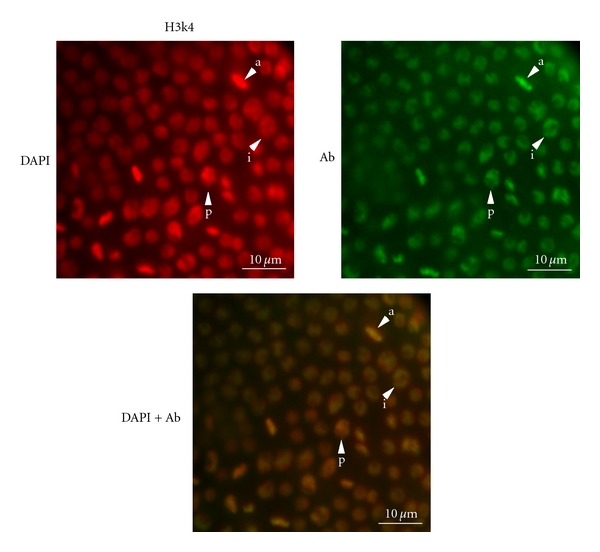
Localized, cell-cycle-dependent H3K4me2 staining in *Daphnia magna* gastrula nuclei. In interphase cells (i), the DAPI staining (red, left) is largely uniform whereas the H3K4me2 staining (green, right) is concentrated at the nuclear periphery producing a yellow-green circle with a red center in the merged image (lower panel). In cells undergoing prophase (p) and anaphase (a) the DAPI and H3K4me2 staining is coincident. Multiple cells in these stages are shown.

The subnuclear distribution of H3K4me2 staining also appears to be dependent on the cell cycle. In interphase nuclei, H3K4me2 staining was the strongest at the periphery of the nucleus and largely excluded from the interior. However, by prophase and metaphase, DAPI and H3K4me2 staining was largely coincident (Figure 3). 

**Figure 4:**
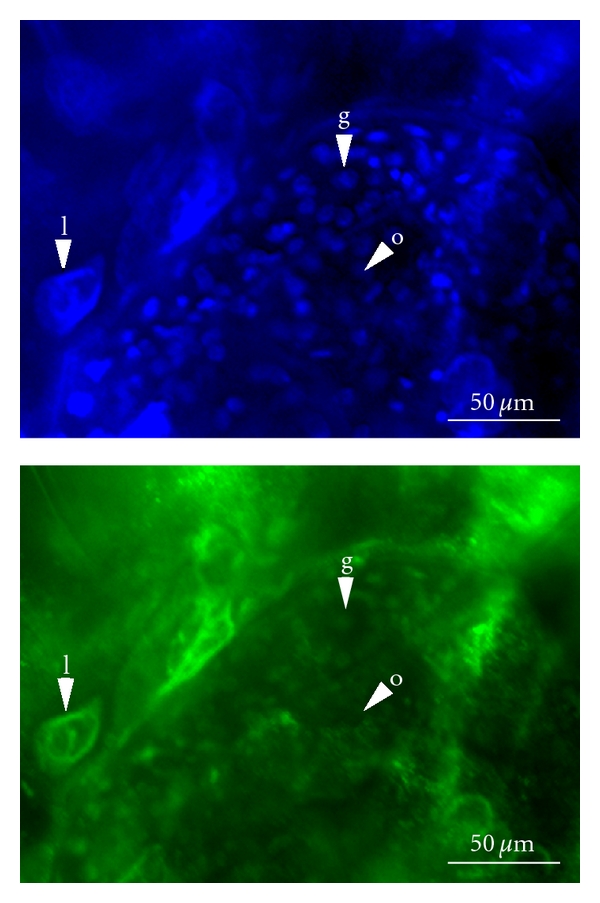
Whole mount immunocytochemistry of *Daphnia magna* ovaries stained with antibodies specific to H3K4me2. The top image shows DAPI staining, which detects all nuclei including the large lipid cell (l), the nuclei of the germarium (g), present in a rosette arrangement, and the nuclei of the developing oocytes and nurse cells (o). The lower image shows the same tissue stained for H3K4me2. The nuclei of the lipid cell and the cells of the germarium are detected. The nuclei of the oocytes and nurse cells are not strongly labelled with this antibody.

To further investigate H3K4me2 distribution in different developmental stages and cell types, antibodies specific to H3K4me2 were used to investigate ovaries from parthenogenetically reproducing females. DAPI staining shows both small cells and bigger cells with large nuclei (Figure 4). The larger cells (Figure 4 (l)) are likely polyploid lipid-containing fat cells [1]. The smaller cells are diploid germ-line cells, either the stem cells in the germarium (Figure 4 (g)) or developing oocytes and their companion nurse cells (Figure 4 (o)). Quartets of these cells remain attached as a result of incomplete cytokinesis in the two preceding divisions and so are clustered [3]. However, histological distinction between the oocyte and nurse cells is not possible until later in development [1, 3]. The staining indicates that H3K4me2 is present in both the germarium cells and somatic fat cells. However, H3K4me2 is either absent from or greatly reduced in the developing oocytes and nurse cells.

## 4. Discussion

While the core epigenetic mechanisms of DNA methylation and histone modification are interrelated [31], organisms vary in the extent of their reliance on each of these mechanisms [32]. For example, in mammals and plants that methylate their genomes extensively, DNA methylation is a key aspect in genomic imprinting. However, in *Drosophila*, which has a much lower level of genomic DNA methylation, genomic imprinting relies primarily on histone modifications and related chromatin-based mechanisms [32, 33]. While *Daphnia* do have DNA methyl transferases and methylate their genome [20, 21], the level of DNA methylation is low, comparable to that of *Drosophila* [21], suggesting that histone modifications may similarly play a larger role in epigenetic regulation. For this reason, we initiated an investigation of histone modifications in *Daphnia*, to our knowledge, the first such investigation. 

We have demonstrated that the epigenetic repertoire of *Daphnia* includes histone modification, represented by the best-characterized methylated and acetylated modifications of histone H3 and histone H4. Histone modifications such as histone 3 trimethylated at lysine 27 (H3K27me3) or monomethylated at lysine 9 (H3K9me) and histone 4 dimethylated at lysine 20 are associated with heterochromatin and are present uniformly throughout the nucleus. In contrast, a modification associated with euchromatin occurs in a reproducible and distinct pattern around the inner periphery of the nucleus. Interestingly, this is the reverse of the usual organization of euchromatin and heterochromatin in the nucleus [34].

Euchromatic and heterochromatic structures influence the transcriptional status of a gene, which is conferred by a dynamic combination of different histone modifications, in conjunction with other epigenetic marks. The nature, abundance, and location within a gene of these epigenetic marks all affect the likelihood of transcription [24–27]. Thus, a single histone modification cannot unambiguously indicate the transcriptional status of a gene or the genome. Further, we are examining these modifications at the level of the nucleus rather than the gene and some of the early embryonic cells examined may not have been transcriptionally active. All of these considerations suggest that the pattern of modifications we see may not be indicative of transcriptional activity. It is, however, interesting that this pattern is the reverse of the canonical arrangement of euchromatin and heterochromatin in the nucleus. Chromosomes typically occupy distinct territories in the nucleus with heterochromatin segregated to the periphery [34]. Nuclei with the reverse organization, including the localization of H3K4me3, which like the H3K4me2 modification studied here is a marker for euchromatin, have been found in the retinal rod cells of nocturnal mammals [35, 36]. This organization has been attributed to selection for increased light transmission under low light conditions. This is unlikely to be the cause of the reversed organization of euchromatin in *Daphnia* embryos. However, as the “reversed” nuclear organization in nocturnal mice arises postnatally and only in rod cells it does demonstrate that genome architecture can be modified by natural selection. Thus, this unusual organization of the nucleus might be more common than previously thought.

This work lays the groundwork for further investigation of histone modifications associated with epigenetic events in the normal life cycle of *Daphnia*, such as the switch from parthenogenetic to sexual reproduction, including the development of males and haploid eggs, and the well described predator-induced epigenetic polyphenisms such as helmets and neckteeth. The external environment plays a role in regulating these key epigenetic events, and some of the genes involved in the signaling pathways by which the external environment influences the epigenome have been identified [11]; it would be interesting to examine the epigenetic status of these genes under varying environmental conditions. Additionally, the gene knockdown technology that has been developed [18, 19] as well as conventional pharmacological inhibition of histone modifying enzymes using trichostatin A or butyrate will allow critical assessment of the role of histone modification in the interplay between the environment and genome in *Daphnia*. Finally, in organisms with conventional sexual reproduction, meiosis and gametogenesis are strictly coupled. However, in *Daphnia* oogenesis occurs with essentially mitotic nuclei. This situation would offer a unique opportunity to discriminate between chromosomal and cellular events in transgenerational epigenetic phenomena such as genomic imprinting.

## References

[1] Zaffagnini F (1987). Reproduction in *Daphnia*. *Memorie dell'Istituto Italiano di Idrobiologia*.

[2] Hiruta C, Nishida C, Tochinai S (2010). Abortive meiosis in the oogenesis of parthenogenetic *Daphnia pulex*. *Chromosome Research*.

[3] Schurko AM, Logsdon JM, Eads BD (2009). Meiosis genes in *Daphnia pulex* and the role of parthenogenesis in genome evolution. *BMC Evolutionary Biology*.

[4] Hebert PD, Ward RD (1972). Inheritance during parthenogenesis in *Daphnia magna*. *Genetics*.

[5] Omilian AR, Cristescu MEA, Dudycha JL, Lynch M (2006). Ameiotic recombination in asexual lineages of *Daphnia*. *Proceedings of the National Academy of Sciences of the United States of America*.

[6] Kleiven OT, Larsson P, Hobaek A (1992). Sexual reproduction in *Daphnia magna* requires three stimuli. *Oikos*.

[7] Olmstead AW, LeBlanc GA (2007). The environmental-endocrine basis of gynandromorphism (intersex) in a crustacean. *International Journal of Biological Sciences*.

[8] Kato Y, Kobayashi K, Watanabe H, Iguchi T (2011). Environmental sex determination in the branchiopod crustacean *Daphnia magna*: deep conservation of a Doublesex gene in the sex-determining pathway. *PLoS Genetics*.

[9] Laforsch C, Tollrian R (2004). Embryological aspects of inducible morphological defenses in *Daphnia*. *Journal of Morphology*.

[10] Agrawal AA, Laforsch C, Tollrian R (1999). Transgenerational induction of defences in animals and plants. *Nature*.

[11] Miyakawa H, Imai M, Sugimoto N (2010). Gene up-regulation in response to predator kairomones in the water flea, *Daphnia pulex*. *BMC Developmental Biology*.

[12] Colbourne JK, Singan VR, Gilbert DG (2005). wFleaBase: the *Daphnia* genome database. *BMC Bioinformatics*.

[13] Colbourne JK, Pfrender ME, Gilbert D (2011). The ecoresponsive genome of *Daphnia pulex*. *Science*.

[14] Watanabe H, Tatarazako N, Oda S (2005). Analysis of expressed sequence tags of the water flea *Daphnia magna*. *Genome*.

[15] Steinberg CEW, Stürzenbaum SR, Menzel R (2008). Genes and environment—Striking the fine balance between sophisticated biomonitoring and true functional environmental genomics. *Science of the Total Environment*.

[16] Keeney S, Tsuchiya D, Eads BD, Zolan ME (2009). Methods for meiotic chromosome preparation, immunofluorescence, and fluorescence in situ hybridization in *Daphnia pulex*. *Meiosis: Volume 2, Cytological Methods*.

[17] Robinson CD, Lourido S, Whelan SP, Dudycha JL, Lynch M, Isern S (2006). Viral transgenesis of embryonic cell cultures from the freshwater microcrustacean *Daphnia*. *Journal of Experimental Zoology Part A: Comparative Experimental Biology*.

[18] Kato Y, Kobayashi K, Watanabe H, Iguchi T (2010). Introduction of foreign DNA into the water flea, *Daphnia magna*, by electroporation. *Ecotoxicology*.

[19] Kato Y, Shiga Y, Kobayashi K (2011). Development of an RNA interference method in the cladoceran crustacean *Daphnia magna*. *Development Genes and Evolution*.

[20] Vandegehuchte MB, Kyndt T, Vanholme B, Haegeman A, Gheysen G, Janssen CR (2009). Occurrence of DNA methylation in *Daphnia magna* and influence of multigeneration Cd exposure. *Environment International*.

[21] Vandegehuchte MB, Lemière F, Janssen CR (2009). Quantitative DNA-methylation in *Daphnia magna* and effects of multigeneration Zn exposure. *Comparative Biochemistry and Physiology*.

[22] Sagawa K, Yamagata H, Shiga Y (2005). Exploring embryonic germ line development in the water flea, *Daphnia magna*, by zinc-finger-containing VASA as a marker. *Gene Expression Patterns*.

[23] Gulbrandsen J, Johnsen GH (1990). Temperature-dependent development of parthenogenetic embryos in *Daphnia pulex* de Geer. *Journal of Plankton Research*.

[24] Jenuwein T, Allis CD (2001). Translating the histone code. *Science*.

[25] Lee JS, Smith E, Shilatifard A (2010). The language of histone crosstalk. *Cell*.

[26] Bannister AJ, Kouzarides T (2011). Regulation of chromatin by histone modifications. *Cell Research*.

[27] Kharchenko PV, Alekseyenko AA, Schwartz YB (2011). Comprehensive analysis of the chromatin landscape in *Drosophila melanogaster*. *Nature*.

[28] Fuchs J, Demidov D, Houben A, Schubert I (2006). Chromosomal histone modification patterns—from conservation to diversity. *Trends in Plant Science*.

[29] Richards EJ, Elgin SCR (2002). Epigenetic codes for heterochromatin formation and silencing: rounding up the usual suspects. *Cell*.

[30] Sims RJ, Nishioka K, Reinberg D (2003). Histone lysine methylation: a signature for chromatin function. *Trends in Genetics*.

[31] Fuks F (2005). DNA methylation and histone modifications: teaming up to silence genes. *Current Opinion in Genetics and Development*.

[32] MacDonald W (2012). Epigenetic mechanisms of genomic imprinting: common themes in the regulation of imprinted regions in mammals, plants, and insects. *Genetics Research International*.

[33] Lloyd V (2001). Parental imprinting in *Drosophila*. *Genetica*.

[34] Cremer T, Cremer C (2001). Chromosome territories, nuclear architecture and gene regulation in mammalian cells. *Nature Reviews Genetics*.

[35] Ragoczy T, Groudine M (2009). The nucleus inside out-through a rod darkly. *Cell*.

[36] Solovei I, Kreysing M, Lanctôt C (2009). Nuclear architecture of rod photoreceptor cells adapts to vision in mammalian evolution. *Cell*.

